# Supplementary figures and images for: Platelet distribution width as a cost-effective marker for sepsis-associated acute kidney injury: A retrospective cross-section study
Source: PLoS One. 2025 May 13;20(5):e0321639. doi: 10.1371/journal.pone.0321639 (PMC12074388; doi:10.1371/journal.pone.0321639)

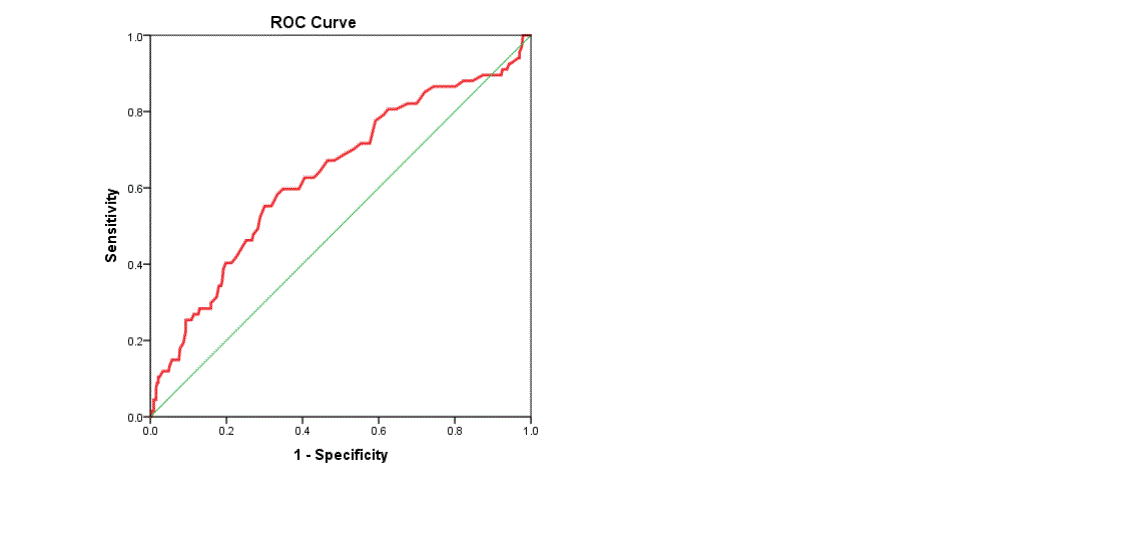

Supplement: S1 Fig — (TIF) [file pone.0321639.s001.tif]
