# Supplementary material for: Platelet distribution width as a cost-effective marker for sepsis-associated acute kidney injury: A retrospective cross-section study
Source: PLoS One. 2025 May 13;20(5):e0321639. doi: 10.1371/journal.pone.0321639 (PMC12074388; doi:10.1371/journal.pone.0321639)
Supplement: S1 Table — (DOCX) [file pone.0321639.s002.docx]

**S1 Table. Spearman's correlation of PDW with disease severity scores and inflammatory markers in sepsis.**

| PDW | SOFA | APACHEⅡ | Charlson's index | PCT | LDH | CRP |
| --- | --- | --- | --- | --- | --- | --- |
| Coefficient | 0.273 | 0.153 | 0.100 | 0.092 | 0.233 | -0.092 |
| P value | <0.001 | 0.008 | .092 | .114 | <0.001 | .113 |
