# Supplementary material for: Platelet distribution width as a cost-effective marker for sepsis-associated acute kidney injury: A retrospective cross-section study
Source: PLoS One. 2025 May 13;20(5):e0321639. doi: 10.1371/journal.pone.0321639 (PMC12074388; doi:10.1371/journal.pone.0321639)
Supplement: S2 Table — (DOCX) [file pone.0321639.s003.docx]

**S2 Table. PDW, SOFA and SOFA-PDW predict ROC curves for S-AKI.**

| Variable | sensitivity（%） | specificity（%） | cutoff value | Yoden's index | *P* value | AUC（95%CI） |
| --- | --- | --- | --- | --- | --- | --- |
| PDW | 56.60 | 71.4 | 12.55 | 0.28 | .000 | 0.696（0.621-0.771） |
| SOFA | 81.1 | 66.8 | 3.50 | 0.48 | .000 | 0.771（0.706-0.837） |
| LDH | 54.7 | 80.0 | 262.50 | 0.35 | .000 | 0.695（0.611-0.780） |
| SOFA-PDW | 88.7 | 58.8 |  | 0.48 | .000 | 0.799（0.739-0.858） |
